# Health Inequities in the Care Pathways for People Living with Young- and Late-Onset Dementia: From Pre-COVID-19 to Early Pandemic

**DOI:** 10.3390/ijerph18020686

**Published:** 2021-01-14

**Authors:** Clarissa Giebel, Caroline Sutcliffe, Frances Darlington-Pollock, Mark A. Green, Asan Akpan, Julie Dickinson, James Watson, Mark Gabbay

**Affiliations:** 1 Department of Primary Care & Mental Health, University of Liverpool, Liverpool L69 3GL, UK; mbg@liverpool.ac.uk; 2 National Institute for Health Research Applied Research Collaboration North West Coast, Liverpool L69 3GL, UK; dickinson27@talktalk.net; 3 Social Care and Society, University of Manchester, Liverpool M13 9PR, UK; caroline.sutcliffe@manchester.ac.uk; 4 Department of Geography and Planning, University of Liverpool, Liverpool L69 3GL, UK; f.darlington-pollock@liverpool.ac.uk (F.D.-P.); Mark.Green@liverpool.ac.uk (M.A.G.); James.watson@liverpool.ac.uk (J.W.); 5 Department of Musculoskeletal & Ageing Sciences, University of Liverpool, Liverpool L69 3GL, UK; asangaedem.akpan@liverpool.ac.uk; 6 Liverpool University Hospitals NHS FT, Liverpool L7 8XP, UK

**Keywords:** young- and late-onset dementia, health inequities, care pathways, health and social care services, community-based services

## Abstract

Background: Little is known about how people with dementia and/or their family carers access health and social care services after a diagnosis. The aim of this study was to explore potential inequalities in care pathways for people with young-onset and late on-set dementia (YOD/LOD), including their family carers, with coronavirus disease 2019 (COVID-19) occurring throughout the course of the study and enabling a comparison between pre-pandemic and COVID-19 times. Methods: People with YOD and LOD with their family carers were recruited via local support groups in the North West Coast region of England. Semi-structured interviews explored the experiences of people with YOD and LOD and family carers on their access to both health and social care services and community-based services. Transcripts were coded by two researchers and analysed using thematic analysis. Fifteen interviews were conducted with seven people with YOD or LOD and 14 family carers between January and March 2020. Some interviews were conducted only with the person with dementia, because they did not have a family carer, and others were conducted only with the family carer, because the person with dementia was in the severe stages of the condition. Results: Four themes emerged from the interviews: (1) Getting the ball rolling: the process of diagnosis; (2) Balancing the support needs of people with dementia and carers; (3) Barriers to accessing support; and (4) Facilitators to accessing support. Inequities existed for both YOD and LOD, with emerging evidence of unequal experiences in accessing care at the beginning of the COVID-19 pandemic. Discussion: People with YOD and LOD and their carers require better support in accessing services after a diagnosis. Greater understanding of the pathways through which inequalities materialise are needed, especially those that might have been disrupted or exacerbated by the COVID-19 pandemic.

## 1. Introduction

Dementia affects over 50 million people worldwide [1], and is a growing public health concern. In the U.K., the number of older people with dementia is projected to double in the next 25 years, with annual costs projected to rise from GBP 23 billion in 2015 to GBP 80.1 billion in 2040 [2]. With the number of people living with dementia rising, care needs to be adequate to support the varying needs of those living with the condition, as well as to be supportive of their family carers. Different subtypes of dementia, such as Alzheimer’s disease, vascular dementia, and frontotemporal dementia, are characterised by different symptoms, including more pronounced deficits with language, memory, behaviours, or specific everyday activities [3,4].

It is estimated that, by 2040, over one million people living with dementia (PLWD) will receive some level of unpaid and/or paid care [2]. Social support services, such as social support groups, having a befriender, attending a day care centre, or engaging in social activities, can all have beneficial effects on the well-being of PLWD and their family carer [5]. However, they may not be suitable for everyone due to personal choices or due to the particular challenges of different types of dementia [6]. When PLWD are not adequately supported via social support services, they are more likely to resort to requiring the use of health care services, including visiting their general practitioner (GP) and going to hospital. Therefore, ensuring equitable access to non-healthcare services is important to ensure the well-being of people with dementia.

Underlying socio-economic factors can be the cause of unmet needs in health and social care. For example, people from more disadvantaged socio-economic backgrounds are less likely to access the care they need. This can be reflected both in terms of medication [7] and access to health and social care services [8,9,10]. Whilst a burgeoning evidence base is exploring individual inequalities in accessing different dementia services, such as only medication, or only care homes, there appears to be little to no evidence exploring the experiences of PLWD and their unpaid carers of accessing different types of care services. Sommerlad and colleagues (2019) [11] reported that people with dementia with everyday functional limitations and/or mental health difficulties, amongst others, had a larger number of hospitalisations. Those from more socio-economically disadvantaged backgrounds also have higher hospitalisation rates. Health inequities, and therefore unjust avoidable differences in health [12,13], are thus common in dementia care. However, there is limited knowledge on the experiences of barriers in accessing healthcare services such as hospitals (or social care services). This is crucial to better understand how barriers could be addressed.

Living with young-onset dementia (YOD), where the age of onset is below 65, and caring for someone with YOD, can also have an effect on the types of services that people with dementia access. Research suggests that post-diagnostic services for dementia are generally not well adapted to the needs of people with YOD and their family carers [14,15]. People with YOD are younger and are likely to want to engage in less sedentary activities, or would like more support surrounding employment. Due to the age difference, people in their 70s or 80s are mostly retired and less likely to have large financial commitments. A recent memory clinic evaluation corroborated these different needs, whilst also showing that the diagnostic process itself was more positive than the lack of support afterwards [16]. Care for people with YOD needs to be addressed differently to care provided for people with LOD. The present non-differentiation in care provision may be causing differences in access.

It is important to acknowledge the changing landscape of care provision that the coronavirus disease 2019 (COVID-19) pandemic and associated public health measures have had on the lives of people with dementia and carers. Since March 2020, the U.K. experienced a nationwide lockdown for several months, with vulnerable and older adults asked to shield until early August [17]. Although evidence is still very sparse, emerging evidence from the U.K. showcases how public health measures have led to a lack of access to social support services, which led to faster deteriorations in dementia and increased carer burden [17]. This is corroborated by international evidence [18]. It is still unclear how the pandemic might have reduced or exacerbated inequalities however, although everyone is likely to have been affected in some form by facing restrictions to accessing support.

The aim of this study was to explore potential health inequalities influencing care pathways for people living with dementia and their family carers. Care pathways not only include health care services, but also community-based activities such as day care centres and social support groups.

## 2. Methods

### 2.1. Participants and Recruitment

People with dementia who had mental capacity to consent, as well as family carers aged 18+ were eligible to take part. The diagnosis of dementia was based on self-reports, and no clinical records were accessed for confirmation. Both current and previous family carers were included in this study. Previous carers are often neglected in research, however have valuable experiences to share. Participants were recruited in a coastal area of the North West region of England via convenience sampling via a local support group through advertising in their monthly newsletter, and by the lead investigator attending monthly group meetings and discussing the study with attendees. Interested PLWD and family carers provided their contact details to the researcher, to be subsequently contacted for consenting and to arrange a date and time for the interview.

### 2.2. Data Collected

Basic demographic characteristics of both PLWD and their family carers were collected, including age, gender, ethnicity, and highest level of education. PLWD also provided information on their living situation (with family member and whether in rented or owned accommodation), age at dementia diagnosis, YOD or LOD, and dementia subtype. Carers also provided information about their relationship with the PLWD.

Semi-structured interviews for people with YOD and LOD and family carers included questions on the types of healthcare services accessed before and since the dementia diagnosis, barriers faced, types of activities engaged with in the community, and experiences of accessing dementia-specific services. People with YOD and their family carers were also specifically asked about their experiences of accessing services relating to their age/the age of the person they are caring for.

### 2.3. Procedure

We obtained ethical approval from the University of Liverpool [Reference ID: 5795], and due to COVID-19 amended the study to be conducted over the phone. Interviews were conducted by research team members trained and experienced in conducting interviews with PLWD and family carers (C.G. and J.W.). PLWD and family carers were interviewed either in their own home or in a quiet room at the University of Liverpool (pre COVID-19 lockdown on March 23), with interviews generally lasting under 60 minutes and one interview lasting 90 minutes. Data were collected between January and April 2020. Interviews were conducted face-to-face before the COVID-19 outbreak and governmental lockdown, and via telephone afterwards. Prior to the interviews, participants provided written informed consent and provided consent over the telephone, which was audio-recorded. Interview data were audio recorded and subsequently transcribed. 

### 2.4. Data Analysis

Data were analysed using inductive thematic analysis [19], focusing on different types of inequalities experienced by people living with dementia and carers. Three research team members (C.G., C.S. and J.W.) with in-depth experience in analysing qualitative data in dementia read through and coded the transcripts separately, and discussed and jointly agreed the generated codes and themes. These identified themes were subsequently discussed with the wider team, including public advisers.

### 2.5. Public Involvement

One family carer for her relative with dementia was a public adviser as part of this study. They attended team meetings, helping to interpret the findings and place them in the context of her lived experiences of caring for someone with dementia. They were reimbursed according to NIHR INVOLVE (2015) [20] guidelines.

## 3. Results

### 3.1. Background Characteristics

Fifteen interviews were undertaken. These included 14 current or former carers discussing themselves and their relatives with dementia, and seven people with dementia talking about themselves. Across the 15 interviews, experiences mostly covered LOD (*n* = 10). People with dementia who took part were on average 70 (±6) years old (range 60 to 76) and most were male (86%). Family carers were on average 67 (±10) years old (range 48 to 85), with the majority being female (64%) and spouses (79%). All participants were White British. Many people with dementia were living with other comorbid conditions such as diabetes, kidney disease and cancer, or other neurological conditions such as Parkinson’s disease. The majority of people with dementia had been offered dementia medication following diagnosis.

### 3.2. Qualitative Findings

Four related themes were identified from the interviews: (1) Getting the ball rolling: the process of diagnosis; (2) Balancing the support needs of PLWD and carers; (3) Barriers to accessing support; and (4) Facilitators to accessing support (see Table 1). Each theme is described in detail below, and participants are identified in the text by their gender, whether they are PLWD or carers, their age, and the interview ID.

#### 3.2.1. THEME 1: Getting the Ball Rolling: The Process of Diagnosis

##### Experiencing Delays and Misdiagnosis

Getting the correct diagnosis depended on GPs’ awareness of dementia, recognition of symptoms and their willingness to do an assessment. Many participants reported delays of several months in receiving an initial dementia diagnosis following a GP consultation, due to being misdiagnosed or having their concerns dismissed. Age appeared to be a factor in diagnosis for some participants. One carer recounted being told that his wife’s symptoms were due to old age, and they received a diagnosis 18 months later following consultation with a new GP. One participant with YOD felt that her symptoms were missed due to her young age. These are illustrated in the quotes below:

*“...she [GP] kept telling me it was old age and I kept asking her to do something and she said no its just old age”*

***(male carer, 85 yrs, 11)***.

*“I think it was because I was quite young when I was diagnosed and I don’t think people recognised that you could have dementia at that age”*

***(female PLWD, 67 yrs, 02)***.

Some participants reported that they had received a timely diagnosis of dementia following their initial GP consultations. However, some participants continued to be concerned about symptoms and behaviours either in themselves or their relatives which involved repeated visits to their GP practices:

*“Backwards and forwards, backwards and forwards, backwards and forwards until eventually he [GP] had no choice but to make an appointment at the hospital”*

***(male PLWD, 71 yrs, 01)***.

The memory service in one part of the city offered a six-week post-diagnostic course. Although some participants described examples of good post-diagnostic support, a few carers who experienced delays in receiving this support spoke of feeling forgotten about. Timeliness of support was a factor for one former carer whose wife had attended a cognitive stimulation group, but *“she was too far gone”* (**male former carer, 74 yrs, 07**) by the time she was diagnosed to gain benefit from it. However, some described examples of good post-diagnostic support, their only concern being its temporary nature:

*“…they taught us all what Alzheimer’s was, 6-week course, they had another course on how to cope with it and then… they had a course for my wife. But unfortunately, all that stopped now, so there isn’t anything now for her to go…, she really enjoyed the last lot of courses which was like interactions and learning”*

***(male carer, 77 yrs, 11)***.

##### Information and Communication

Both carers and people with dementia spoke about their initial feelings of fear, anxiety, and shock on receiving a dementia diagnosis and their need for information and guidance. Whilst a few participants reported receiving no information initially, there appeared to be variation in the range and type of information that was provided. One carer complained about the paucity of information given following her father’s diagnosis, whilst another carer praised the care co-ordinators but found the information provided was narrow in scope, as illustrated in the quotes below:

*“…if you look to the right there’s a bookcase, there’s a leaflet and that was it, very unprofessional and I was quite flabbergasted by that to be honest with you”*

***(female carer, 48 yrs, 13)***.

*“…eventually the care coordinators contacted us and they were good, and she gave us a list of things that we could take part in… but it wasn’t a complete list… it was, it was really only what they do at [the memory clinic] so the other things that we do, we’ve found out ourselves”*

***(female carer, 64 yrs, 08)***.

#### 3.2.2. THEME 2: Balancing the Needs of PLWD and Carers

##### Accessing Support

The opinions of participants of the services and support that they accessed were coloured by their different perspectives. Some carers preferred groups that were organised and attended only by their peers. There was a perception by some of a gender imbalance in groups, with men being under-represented which led to the setting up of a men-only carers group to encourage attendance. A few participants spoke about perceived inequities between people living with dementia and carers in respect of whose viewpoint took priority. The quotes below contrast the views of a person with dementia who felt carers dominated the service user forum he attended, with that of a carer who believed that her views were deemed less important:

*“…there’s about 10 of us at the most with Alzheimer’s, or dementia. The rest out of 40 people are carers or past carers and the past carers tend to dominate the meeting and what takes place. The small group of us that can put our points of view forward tend to be shouted down”*

***(male PLWD, 67 yrs, 03)***.

*“…the views of people who are carers seem to be less valuable in some people’s eyes than those of the people who are actually living with dementia”*

***(female carer, 67 yrs, 04)***.

Some carers spoke about the difficulties of finding alternative care for their relatives with dementia to allow them to attend peer support groups. This was especially true for people in the more advanced stages of dementia who could not benefit from attendance. A few carers reported that they had occasionally left their relative alone at home in order to attend a group. One carer wanted to attend a cookery course but there was nowhere to accommodate his wife at the carer’s centre. Another carer needing a few hours every now and then to be able to attend medical appointments for themselves described the difficulties encountered in daily life due to a lack of consistent respite care provision for their relative with dementia. These are illustrated in the quotes below:

*“…that’s the one trouble with [name of charitable organisation] they’ve got...a carers centre, but you can’t take the person you care for...which I think is a bit of a problem”*

***(male ex-carer, 74 yrs, 07)***.

*“All I wanted was to be able to go to the dentist or doctor’s appointment… she’ll have to come with me and I had to have a quiet word with the receptionist and say could you keep an eye on my wife while I go up and have my teeth done”*

***(male carer, 85 yrs, 10)***.

##### Experiences of People Affected by YOD

People diagnosed with YOD and their carers spoke about a perceived lack of services aimed at people with early onset dementia, from which they would benefit, and described how existing dementia services appeared to be less pertinent or convenient for them. One consequence of living with dementia at a younger age was belonging to a working-age generation with competing care responsibilities. This was voiced by a carer who worked and also cared for a grandchild so could not take her husband to a young-onset group, but he could not attend alone. Some YOD participants and carers spoke about the slow pace of the post-diagnostic support group and of the activities favoured by the older group members such as bingo and old films, and the general lack of groups specifically for those with early onset dementia. These comments and differential needs were indicative of the age gap between YOD and LOD These are illustrated in the quotes below:

*“not everybody’s the same and there is a local group who meet up for coffee, they’re probably not the people that we, these people are all very nice but probably they’ve had a different life experience than us.”*

***(male carer, 70yrs YOD, 02)***.

*“…at the moment there’s not a lot they [social/community groups] can do for us, because there’s no early onset groups...there’s no early onset meeting places, so you have to go and do something yourself”*

***(female carer, 57 yrs YOD, 05)***.

#### 3.2.3. THEME 3: Barriers to Accessing Support

##### Costs of Care

There was evidence of inequalities in accessing services based on levels of personal finances, whereby out of pocket costs could influence access to care and support. This is illustrated in the quote below from a carer who was concerned of the long-term cost that would be incurred if his wife living with dementia was to regularly attend a day care centre:

*“there was a suggestion of a day centre which is not too far away from us…we had the assessment and they were quite happy for her to go in there for one day a week…I asked how much and they said...that the council do that…they said well you will have to answer questions about her financial situation. But… £20 a day, and really I can’t afford to pay £20 a day”*

***(male carer, 83 yrs, 12)***.

Younger carers still in employment and whose partners were living with YOD spoke about problems encountered in accessing affordable care for their relatives, or having to rely on other family members to provide informal care whilst out at work. Another recounted the upset caused by the delay in receiving funding for continuing healthcare for her husband, illustrated in the quote below:

*“They said they’d take him, so then, took 6 weeks to get the funding through but there’s not a lot you can do about that. That was the worse time really because you knew it was going to happen, you knew you were going to get the funding but just waiting you know”*

***(female carer, 65 yrs YOD, 09)***.

##### Variation in Service Availability

Another barrier to accessing services, expressed by a few participants, was the variation in the availability and consistency of services across Liverpool. There had been a city-wide withdrawal of dementia-specific services by a large national organisation reportedly due to the loss of council funding which highlighted the tenuous nature of commissioning of third sector organisations. A lack of consistent secondary health care support was a concern raised by some participants regarding the care navigator service and Parkinson’s nurse service both viewed as being understaffed, resulting in infrequent patient contact as illustrated in the quote below:

*“They are constrained by the fact that they’ve only got three care navigators, supposed to be taking on a few more so that might improve. It’s just a question of …getting the care navigator to ring me up a bit more frequently”*

***(male PLWD, 67 yrs, LOD, 03)***.

Some carers noted that a lack of respite care restricted them from attending classes provided specifically for carers. Others spoke about the problems in accessing in-home respite (in the form of a sitting service). The city council issued care vouchers to “purchase” alternative care and provide carers with a break. A few carers described the difficulty of redeeming vouchers for sitting services that were not available in their area:

*“I’ve had some care vouchers, that somebody could come in and sit with her for up to 2 hours, while I went out to do things but when I checked, it was just three or four places, I rang, [they] said ‘oh no we don’t do your area’”*

***(male carer, 83 yrs, 12)***.

##### Transport

A significant barrier to accessing support services was related to a perceived lack of available transport. Many of those living with dementia had given up driving and relied upon the carer to take up driving duties. For those without access to a car, however, this meant difficulties in accessing support groups that were not in their immediate locality. Some carers noted the inconvenience caused by travelling across the city and the cost incurred to attend medical appointments and services, or the problems encountered when they did not have access to a car, as illustrated in the quotes below:

*“Well if I haven’t got my car for any reason…because we were going to [locality of memory clinic] for ages and when I was having my eye done in the first place...and it was costing us an absolute mint”*

***(male carer, 77yrs, 11)***.

*“It’s just very very difficult getting support you know to get her [sister with dementia] there…, well it’s transport, I don’t drive so we have to get the bus and I’ve got nobody else you know I don’t have any children who can stand in and you know give her a lift”*

***(female carer, 67 yrs, 15)***.

##### Geographical Inequities

There were also perceived geographic inequities in service provision for people with dementia. The lack of a post-diagnostic group in the northern area of the city was noted by some participants. People living some distance away from services provided in the south of the city meant they had to travel further to access services in the other relatively resource-rich locality, as illustrated in the quote below:

*“…our end of the city was and still is to quite a large extent not well served by [name of NHS Trust] and that’s not just my opinion… Because [Service User Reference group]…, membership, the majority of the members live in the south end of the city because it sort of grown up around people who went to [the memory clinic] so we didn’t get a post diagnostic group even though I was pushing for it and in the end I said well we’ll come to [the memory clinic] because you know I can drive”*

***(female carer, 67 yrs, 04)***.

##### Effect of Covid-19 Pandemic

Some interviews were conducted in the weeks following the COVID-19 outbreak, but prior to shielding advice given to clinically vulnerable groups [21] and the subsequent lockdown of households. Participants who were aware of news coverage were concerned about their relatives’ susceptibility to the disease, their ability to continue to care, and the safety and availability of services. Some were concerned about the impact of COVID-19 on their ability to care if they themselves contracted the virus, and the difficulties in undertaking care tasks such as delivering meals and keeping their relative clean whilst staying at a safe distance. Another carer, whose sister with dementia lived alone, commented on her sister’s inability to grasp the concept of lockdown and of social distancing when going outside.

Many non-statutory and voluntary groups had begun to close, and carers voiced concerns about the effect of losing care services and providing family support in place of formal services. Participants’ concerns centred mainly around fears of isolation and loneliness. In one example, a carer remarked that her father, who lived alone, would lose daytime company if his day care centre closed, and the family would need to provide informal care for him. Another carer expressed a fear that staff absenteeism would threaten continued delivery of home care for his wife. These are illustrated in the quotes below:

*“…so that [formal care provision] hasn’t stopped at the moment thank god and obviously I think…when that does I think well there’s no other option he [father living with dementia] will probably just have to come and live with us”*

***(female carer, 48yrs, 13)***.

*“So eventually I think when at the height of this we’ll be isolated a bit because they’re all stop, that’s stopping, the carers association is stopping so they’ll all stop eventually won’t they?”*

***(male carer, 77 yrs, 11)***.

#### 3.2.4. THEME 4: Facilitators to Accessing Support

##### Timely Diagnosis and Referral

Whilst a number of participants recounted stories of delays in diagnosis and follow-up support, those that received a speedy referral praised the post-diagnostic support that was delivered. This access to information and support at an early stage was regarded as beneficial in helping them to seek assistance and in decision-making for the future as illustrated below:

*“I think it’s about six weeks... where you go with the person who’s got dementia and like their family or the carer and talk as a group about what things to expect, how to handle things…it also looks at the legal side so you know it gives us advice on getting power of attorney for both health and finances, tells us what direction to go in”*

***(female carer, 55 yrs YOD, 14)***.

##### Being Proactive

In many cases, participants found that a solution to a perceived lack of information or service was to independently set up their own groups or join peer-led groups. This was to some extent a response to the loss of a large national dementia charity from the city and subsequent closure of their varied support groups, and the loss of a day care centre. Some used social media, joining WhatsApp or Facebook groups set up by informal carers.

*“She set up a nice little group on Facebook for people who are in similar situations so you could at least go and talk and say, well do you know what to do in this situation, and I met a couple of people who’d come from that same place where I’d been, the same unfortunate process they’d had”*

***(female carer, 48 yrs, 13)***.

##### Joined-Up Services

Dementia care provision seemed to work well were there was evidence of joined-up services or multidisciplinary team working. Many participants praised the support of allied professionals such as Admiral Nurses, clinical psychologists, social workers, and non-clinical staff such as handyman services. Having a single point of access and continuity of care was also valued by participants, so that they knew where and who to contact if they needed assistance. Multidisciplinary working was regarded as beneficial, as illustrated in the quotes below:

*“…because of occupational therapists, CPNs, the whole gambit really, psychologists. I’ve got a better understanding of what and people have bent over backwards I think to try and teach me.”*

***(male PLWD, 71 yrs, 01)***.

*“…the only person who’s been absolutely wonderful to us has been the Admiral Nurse we got…And that girl’s been fantastic, she’s put us in touch with loads of different places, just fantastic, even down to putting us in touch with the handyman services, so any minor repairs my dad had around the house their handyman would come, all free of charge”*

***(female carer, 48 yrs 13)***.

*“… and then we have a point of contact [at the memory clinic] our nurse is called A (name of nurse) and she’s fantastic so she [is] at the end of the phone if I ever need to...”*

***(female carer, 55 yrs YOD, 14)***.

## 4. Discussion

This exploratory study highlights inequalities in care provision in both YOD and LOD, and provides some emerging evidence on how the COVID-19 pandemic was beginning to affect dementia care.

Inequalities were noted across the care pathways of dementia, from diagnosis and the time it took to receive a diagnosis, to receiving any form of social support services post-diagnosis. This supports previous evidence on individual aspects of the care pathways, such as the diagnosis process, or accessing services [22,23], and adds important knowledge on the inequitable access of care across the spectrum of dementia. Not only did people with LOD and their carers face difficulties, but inequalities were equally, and possibly even more, evident in people with YOD and their carers. Literature highlights how people with YOD and carers feel that services are not tailored to their needs—particularly to their age [7]. This was reflected in our interviews, with many participants highlighting the need for the better tailoring of services.

In addition to services and their format being considered a hindrance to usage, geographical location was also stated as an example of inequitable access. With previous evidence supporting the impact of place of residence (i.e., rural versus urban) on accessing dementia care [24,25], we found evidence of inequalities happening within cities and often on a far smaller scale. Participants highlighted how particular types of dementia care were only located within one part of the city region, placing additional burden on the provision of care on those with limited access or opportunities to travel. Addressing the care gap for both patients and their carers is a feasible opportunity for policy to improve the quality of care.

Even when services are available and might be suited to the needs of someone, it is often dependent on the unpaid carer to ensure access to services. Carers are mostly reliant on peers or on their own proactiveness to identify and reach out to those services. Where carers remain unaware of particular services, this increases the care burden for them. Carers in England alone provide the equivalent of over GBP 11 billion a year in unpaid care [26], a service which can have significant impacts on their own health and wellbeing. Carers are likely to provide this high level of support, in light of the penalisation of PLWD and carers who have been diligent with and saving their money throughout their life, and by thus being over a certain threshold of income/savings, would have to pay for external additional care, despite their limited income. In contrast, PLWD and carers who have not been saving are also at the lower side of the socio-economic status, and will receive financial support from the government and council to pay for their external care support. This is a significant inequality in itself. Our findings therefore suggest that carers need help to better support the person they care for, and for their own mental well-being. Better support for carers is further likely to benefit patients.

With interviews conducted between January and March 2020, the early effects of the COVID-19 pandemic on accessing services already became apparent in some interviews conducted towards the end of the study. Carers were starting to become concerned about the lack of continued support provision, which has been subsequently evidenced in U.K.-wide research on the detrimental impact of COVID-19 public health measures on social support provision in dementia [16]. Although critical work is emerging on the impacts on care homes during the pandemic [27,28], research must not neglect the large proportion of PLWD living in the community who have also been impacted by COVID-19 indirectly within their own home. This is usually due to reductions in social support and social care. It remains unclear whether the pandemic has affected access to services equally amongst people with dementia and carers, or whether certain inequalities have been exacerbated. However, inequalities may be introduced; it is emerging that most services are now provided remotely and via digital platforms [16]. Unequal access to or use of either a computer or appropriate smartphone, whether due to financial barriers or a lack of technical expertise, complicates healthcare provision where this has moved to digital platforms. Indeed, many older adults and those with chronic health conditions do not have the skills or understanding of how to work Zoom or Skype for remote support [22]. This requires continued attention throughout the pandemic, and post-pandemic.

Whilst we collected data from the spectrum of dementia, with both YOD and LOD represented, as well as different dementia subtypes, our study is subject to some limitations. Data were collected from one area in the North West of England, and are therefore not representative across the country. However, this was an exploratory study which has highlighted numerous inequalities in care, providing reasons to explore this issue more widely. This lack of representation is also reflected in the ethnic background of participants within this study; all participants were White British. This might be linked to our recruitment strategy and the representation of people with dementia and carers within the social support group from which we recruited. This may have also resulted in no ethnic minority representation in the study. Existing evidence highlights various inequalities that people with dementia and carers from ethnic minority backgrounds are facing [10,29]. Recruiting from a social support group also limited the experiences that people with dementia and carers might have. Everyone was receiving some level of support; therefore, those who are not aware of how to access services or do not wish to engage in services were not captured in this study. However, many participants shared their experiences of how they struggled to access care at the beginning of the dementia process, prior to accessing the support group.

## 5. Conclusions

Both people with YOD and LOD, as well as their family carers, face a number of inequalities in accessing health and social care services, ranging from diagnosis to post-diagnostic support and care. Whilst people with YOD and their carers face particular challenges, new pathways resulting in disadvantages have emerged since the pandemic outbreak. Therefore, it is important to address these inequalities both in the light of the pandemic but also for the post-pandemic time, to ensure that each single PLWD and carer is able to access the care they need at the right time. This may be achieved, for example, by involving people with YOD and LOD and carers in the discussions of how services should be developed and delivered.

## Figures and Tables

**Table 1:** Themes from participant interviews.

Main Theme	Sub-Themes
1. Getting the ball rolling: the process of diagnosis	Experiencing delays and misdiagnosis
	Information and communication
2. Balancing the needs of PLWD and carers	Accessing support
	Experiences of people with YOD
3. Barriers to accessing support	Costs of care Variation in service availability Transport Geographical inequities Effect of Covid-19 pandemic
4. Facilitators to accessing support	Timely diagnosis and referral Being proactive Joined-up services

## Data Availability

The data presented in this study are available on request from the corresponding author. The data are not publicly available due to ethical reasons.
